# An Applicable Machine Learning Model Based on Preoperative Examinations Predicts Histology, Stage, and Grade for Endometrial Cancer

**DOI:** 10.3389/fonc.2022.904597

**Published:** 2022-05-30

**Authors:** Ying Feng, Zhixiang Wang, Meizhu Xiao, Jinfeng Li, Yuan Su, Bert Delvoux, Zhen Zhang, Andre Dekker, Sofia Xanthoulea, Zhiqiang Zhang, Alberto Traverso, Andrea Romano, Zhenyu Zhang, Chongdong Liu, Huiqiao Gao, Shuzhen Wang, Linxue Qian

**Affiliations:** ^1^Department of Ultrasound, Beijing Friendship Hospital, Capital Medical University, Beijing, China; ^2^Department of Radiation Oncology (Maastro), GROW School for Oncology and Reproduction, Maastricht University Medical Centre+, Maastricht, Netherlands; ^3^Department of Obstetrics and Gynecology, Beijing Chao-Yang Hospital, Capital Medical University, Beijing, China; ^4^Department of Obstetrics and Gynecology, GROW-School for Oncology and Developmental Biology, Maastricht University Medical Centre, Maastricht, Netherlands

**Keywords:** machine learning, endometrial carcinoma, diagnosis, prediction, random forest, preoperatively

## Abstract

**Purpose:**

To build a machine learning model to predict histology (type I and type II), stage, and grade preoperatively for endometrial carcinoma to quickly give a diagnosis and assist in improving the accuracy of the diagnosis, which can help patients receive timely, appropriate, and effective treatment.

**Materials and Methods:**

This study used a retrospective database of preoperative examinations (tumor markers, imaging, diagnostic curettage, etc.) in patients with endometrial carcinoma. Three algorithms (random forest, logistic regression, and deep neural network) were used to build models. The AUC and accuracy were calculated. Furthermore, the performance of machine learning models, doctors’ prediction, and doctors with the assistance of models were compared.

**Results:**

A total of 329 patients were included in this study with 16 features (age, BMI, stage, grade, histology, etc.). A random forest algorithm had the highest AUC and Accuracy. For histology prediction, AUC and accuracy was 0.69 (95% CI=0.67-0.70) and 0.81 (95%CI=0.79-0.82). For stage they were 0.66 (95% CI=0.64-0.69) and 0.63 (95% CI=0.61-0.65) and for differentiation grade 0.64 (95% CI=0.63-0.65) and 0.43 (95% CI=0.41-0.44). The average accuracy of doctors for histology, stage, and grade was 0.86 (with AI) and 0.79 (without AI), 0.64 and 0.53, 0.5 and 0.45, respectively. The accuracy of doctors’ prediction with AI was higher than that of Random Forest alone and doctors’ prediction without AI.

**Conclusion:**

A random forest model can predict histology, stage, and grade of endometrial cancer preoperatively and can help doctors in obtaining a better diagnosis and predictive results.

## 1 Introduction

Endometrial carcinoma (EC) represents the sixth most common malignant tumor worldwide (1). In 2020, the number of new cases of endometrial cancer was 417,367, and the number of new deaths was 97,370 (1). This may be due to increased obesity, aging, and physical inactivity (2, 3). Endometrial carcinoma occurs most commonly in postmenopausal women (4). The first symptom is often abnormal vaginal bleeding. Transvaginal ultrasound is an effective examination to evaluate the presence of endometrial carcinoma, besides pelvic and physical examination (2, 5). A histopathology diagnosis is commonly assessed by dilation and curettage (D&C) or endometrial biopsy before surgery. However, the preoperative endometrial biopsy and final diagnosis are not completely consistent with only a moderate agreement rate on grade, especially for grade 2 tumors (2). In addition, other serological and imaging tests are routine tests for the diagnosis of endometrial carcinoma (2, 3).

With the development of computer science, clinical decision support systems (CDSSs) are being developed. A CDSS is defined as a system that enhances clinical information and medical knowledge to help doctors and nurses with clinical decisions for better health care (6). CDSS is a major subject of medical artificial intelligence (AI). CDSS can be used pre-diagnosis (prepare diagnoses), during diagnosis (review and filter diagnoses), and post-diagnosis (predict future events).

However, there are no studies that use an AI model to predict histology, stage, and grade for endometrial carcinoma based on the preoperative examinations. Such an AI model can be a part of an endometrial cancer CDSS to improve the efficiency of doctors, reduce the rate of misdiagnosis, and improve the quality of health care.

Machine learning (ML), a type of AI (7), is widely used in medical fields, such as anatomy, medical diagnoses, and brain-machine interfaces (8). In 2022 Otani et al. proposed an ML-based classifier to predict the EC risk from the multiparametric magnetic resonance images (MRI) (9). And, in 2021, Nakajo et al. proved that an 18F-FDG PET-based radiomic analysis using a machine learning approach may be useful for predicting tumor progression and prognosis in patients with endometrial cancers (10).

In this study, we used ML to build three models to predict histology (type I and type II), stage, and grade for endometrial carcinoma to quickly give a diagnosis and assist in improving the accuracy of the diagnosis, which can help patients receive timely, appropriate, and effective treatment.

## 2 Methods

### 2.1 Study Subject

This study used a retrospective database of preoperative examinations in patients with endometrial carcinoma who were first treated in the Department of Obstetrics and Gynecology at Beijing Chaoyang Hospital, Capital Medical University, from January 2000 to April 2014. Inclusion criteria were as follows: (1) undergoing surgical treatment at Beijing Chaoyang Hospital, (2) confirmation of endometrial carcinoma by postoperative pathology, (3) without neoadjuvant chemotherapy and hormone therapy, (4) all treatments have been completed, (5) complete clinical-pathological data. The case exclusion criteria were: (1) presence of primary malignant tumors of other organs, (2) metastatic cancer caused by malignant tumors of other organs, (3) not the first-time surgical treatment at Beijing Chaoyang Hospital, (4) with neoadjuvant chemotherapy and hormone therapy, (5) incomplete clinical-pathological data. The obtained data included age, BMI, childbirth history, preoperative serum tumor markers, imaging results, histopathology diagnosis after D&C, hypertension, diabetes, menopause, symptoms, postoperative histology, stage based on the 2014 International Federation of Gynecology and Obstetrics (FIGO) staging system (11), and grade. Ethics approval for this research was given by the Beijing Chaoyang Hospital, Capital Medical University.

### 2.2 Data and Machine Learning Algorithms

A total of 16 features mentioned above were used for the development of the classification models.

For data preprocessing, first, we transformed semi-structured and unstructured features such as preoperative serum tumor markers and imaging results into structured features. Then, we normalized the continuous variables such as age and BMI into 0 to 1.

In this study, we trained and compared three classifiers, including logic regression(LR) (12), random forest (RF) (13), and a deep neural network(DNN) (14). The DNN is based on the extension of the perceptron: a neural network with many hidden layers. Random forest is an ensemble algorithm (Ensemble Learning), which belongs to the Bagging algorithms. By combining multiple weak classifiers, the result is voted or averaged, so that the result of the overall model has higher accuracy and generalization performance.

The DNN model was composed of two fully connected layers which have a Rectified Linear Unit (ReLU) activation function to increase the nonlinearity of the neural network model and dropout layers with the rate of 0.5 to avoid over-fitting and one fully connected layer without activation function. The cross-entropy loss was used to guide the training process by using a stochastic gradient descent (SGD) optimizer with a 0.0002 learning rate. The random forest included 100 decision trees.

The classification models were trained and tested with the selected features to predict the histology, stage, and grade of endometrial cancer. For model training, we trained and validated the model 100 times (RF, LR) and 10 times (DNN) repeating random sampling verification. We split the dataset into training and testing datasets with a ratio of 7:3 in each validation. Then we used the Synthetic Minority Oversampling Technique (SMOTE) method in the training set for over-sampling, which adds artificially simulated new samples to the data set to decrease the influence of imbalanced data.

To evaluate the performance of the classification models, we calculated the Area Under the Curve (AUC) and the accuracy.

In addition, we also investigated whether the AI algorithms can play a role in the accuracy and speed of the doctor’s diagnosis. We generated four test sets for doctors with 40 patients, half of the patients with an AI prediction class and its possibility, and the other half of the patients without any assistance. Then we sent the test sets to obstetric oncologists to measure the AUC, accuracy, and the time consumption for predicting the disease category with and without AI assistance. The function of accuracy is shown below.


$$accuracy=\frac{TP+TN}{TP+TN+FP+FN}$$

TP, True Positive; TN, True Negative; FP, False Positive; FN, False Negative.

Data pre-processing and machine learning models were implemented within Python 3.8, and scikit-learn 0.24 and PyTorch 1.10 packages.

Comparison of Different Models

The comparison of accuracy between models was performed by using the two-way ANOVA test in GraphPad Prism.

## 3 Results

### 3.1 Clinical Information of Cases

A total of 344 endometrium cancer cases were reviewed and collected. Of these, 14 cases were excluded because of 70% or more of missing clinical data. As there was only one undifferentiated case, this category could not be tested because the test sample would be 0. Therefore, 329 cases were enrolled into the train and test. The mean age was 56 (range 28-83) years old (**Table 1**). The mean BMI was 26.87±4.43. Among these cases, 86.3% of the patients were type I EC. Most (75.7%) of the cases were FIGO stage I and 31 cases were grade (G) 1, 114 cases were G2, 38 cases were G3, and 17 cases were unknown.

**Table 1:** Clinicopathological data of patients with endometrial cancer.

Features	Frequency (%)
	N=329
Age, mean (range)	56 (28-83)
BMI, mean±SD	26.87 ± 4.43
Hypertension	
+	144 (43.8)
–	184 (55.9)
Unknown	1 (0.3)
Diabetes	
+	71 (21.6)
–	256 (77.8)
Unknown	2 (0.6)
Gestation	
+	312 (94.8)
–	17 (5.2)
Parturition	
+	301 (91.8)
–	28 (8.5)
Menopause	
+	192 (58.3)
–	13 (4.0)
Unknown	124 (37.7)
Histology	
type I	284 (86.3)
type II	45 (13.7)
FIGO Stage (2009)	
I	249 (75.7)
II	28 (8.5)
III	42 (12.8)
IV	10 (3.0)
Differentiation	
G1	31 (37.7)
G2	114 (45.6)
G3	38 (11.6)
Unknown	17 (5.2)

G, grade; SD, standard deviation; FIGO, the international federation of obstetrics and gynecology.

### 3.2 Comparison of the Models for the Prediction

#### 3.2.1 Histology

The AUC and accuracy score of the LR were 0.69 (95% CI=0.67-0.70) and 0.74 (95%CI=0.72-0.75). The AUC and accuracy score of RF were 0.69 (95% CI=0.67,0.70) and 0.81 (95%CI=0.79-0.82). The AUC and accuracy score of DNN were 0.60 (95% CI=0.54-0.65) and 0.83 (95% CI=0.75-0.90). The LR and RF algorithms had a similar score which was significantly better (p<0.05) than DNN.

#### 3.2.2 Stage

The AUC and accuracy score of the logistic regression were 0.56 (95% CI=0.54-0.59) and 0.42 (95% CI=0.41-0.44). The AUC and accuracy score of the random forest were 0.66 (95% CI=0.64-0.69) and 0.63 (95% CI=0.61-0.65). The AUC and accuracy score of DNN was 0.48 (95% CI=0.46-0.51) and 0.78 (95% CI=0.71,0.84). The RF was significantly better than LR and DNN.

#### 3.2.3 Grade

The AUC and accuracy score of the LR were 0.61 (95% CI=0.60-0.62) and 0.36 (95% CI=0.35-0.38). The AUC and accuracy score of RF was 0.64 (95% CI=0.63-0.65) and 0.43 (95% CI=0.41-0.44). The AUC and accuracy score of DNN were 0.47 (95% CI=0.45-0.50) and 0.43 (95% CI=0.40-0.45). The LR and RF algorithms have a similar score significantly better than DNN.

### 3.3 Performance Comparison Between ML Model, Doctors’ Prediction, and Doctors With the Assistance of AI

The result of the doctors’prediction is shown in **Table 2**. The average accuracy for histology was 86% (with AI) and 79% (without AI), respectively. The average accuracy for the stage was 64% and 53%, respectively. The average accuracy for differentiation was 50% and 45%, respectively. The time consumption for each patient to make a decision was 29.25 s (with AI) and 28.75 s (without AI), respectively. For type and stage diagnosis, the AI model can improve 6% and 10% of a doctor’s accuracy. But the accuracy decreases 7% for the differentiation diagnosis. The average time consumption with AI was 10 s longer than that without AI, though the AI model only cost 3 ms to predict one patient.

The comparison of a doctor’s prediction with and without AI assistance is shown in **Figure 2**. Compared to LR (**Figure 2A**), the accuracy of doctors’ prediction with AI is higher than that of LR and doctors’ prediction without AI among histology, stage, and grade. The comparison with RF (**Figure 2B**) also showed similar results. However, the accuracy of the DNN’s prediction of the stage was significantly higher than that of doctors’ prediction with and without AI assist (**Figure 2C**). But the accuracy of the combination of doctor and AI was relatively better as a whole.

## 4 Discussions

Endometrial cancer is a relatively common gynecological tumor. The development and application of AI in the medical field has gradually generated significance and value. This study built AI models to predict histology, stage, and grade of EC. Besides the prediction of AI models, we also compared the AI models, doctors’ predictions, and doctors’ predictions assisted by the AI model.

**Figure 1:**
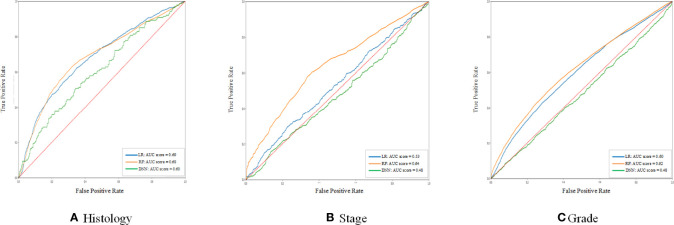
The ROC curve of the histology stage and grade between different models. **(A–C)** shows the ROC curve and AUC score of three different models for histology, stage, and grade prediction, respectively.

From the point of AUC alone, LR and RF models perform better in the prediction of histology and grade. RF is better in the prediction of the stage (**Figure 1**). If only accuracy is considered, DNN and RF models work well in the prediction of histology and grade (**Table 3**). In the real world, not every patient can complete all examinations. In this way, the patients with missing values were also included in the dataset. However, compared with RF and DNN, the LR is sensitive to missing values, which means the missing values will significantly influence the performance of LR (15). On the other hand, DNN with hidden layers has more capability to learn from nonlinear and complex relationships. But it has higher requirements for the sample size of training data than LR and RF (16).

**Table 3:** The AUC score and accuracy of three ML models for histology, stage, and grade prediction.

Model	Histology		Stage		Grade	
	AUC	Accuracy	AUC	Accuracy	AUC	Accuracy
**LR**	0.69 (0.67-0.70)	0.74 (0.72-0.75)	0.56 (0.54-0.59)	0.42 (0.41-0.44)	0.61 (0.60-0.62)	0.36 (0.35-0.38)
**RF**	0.69 (0.67-0.70)	0.81 (0.79-0.82)	0.66 (0.64-0.69)	0.63 (0.61-0.65)	0.64 (0.64-0.65)	0.43 (0.41-0.44)
**DNN**	0.60 (0.54-0.65)	0.83 (0.75-0.90)	0.48 (0.46-0.51)	0.78 (0.71-0.84)	0.47 (0.45-0.50)	0.43 (0.40-0.45)

LR, Logic Regression; RF, Random Forest; DNN, Deep Neural Network; AUC, Area Under the Curve.

Taking into account the above reasons, the RF model was relatively better than other models, so RF was used to assist doctors.

**Figure 2:**
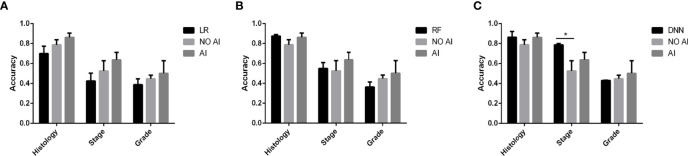
The accuracy comparison between doctors with and without AI assistance and AI in predicting stage and grade. **(A–C)** shows the different AI assistance models. * Indicates P < 0.05

The doctor’s clinical experience combined with the assistance of AI increases the accuracy of histology, stage, and grade (**Table 2**). The main reason is that doctors analyze the highly relevant features of the disease (such as BMI, D&C, imaging, etc.) based on their clinical experience and draw conclusions, while the algorithm learns the influence weights of different features according to the distribution of training data, and more accurate judgments can be obtained for some patients who are not obvious in the preoperative features. Overall, the accuracy of doctors with AI assistance is relatively the best choice among the histology, stage, and grade whether compared to AI alone or doctor alone (**Figure 2**). Therefore, the judgment of the doctor with the RF assistance is the best choice.

**Table 2:** Comparison of doctors’ predictions with and without AI assistance.

Project	Without AI (accuracy %)	With AI (accuracy %)
Histology	79	86
Stage	53	64
Differentiation	45	50

AI, artificial intelligence.

The accuracy of grade and stage is not that high, and the AUC is also relatively low. The reasons can be: 1. The pathological results of preoperative curettage are not completely accurate, and there are false negatives (3); 2. The staging of endometrial cancer is the clinicopathological stage, the determination of staging requires a combination of preoperative conditions, staged surgery and postoperative pathology, as well as grade, but the aim of this study is the preoperative diagnosis, so only preoperative features are given to AI models and doctors, and the intraoperative and postoperative characteristics were not included. Despite this, the AUC of RF is greater than 0.6 among histology, stage, and grade, so it has predictive value, especially given that it is only based on preoperative features.

Furthermore, in the past years, there is general agreement that AI may assist physicians in making better clinical decisions. This technology can provide additional information to help doctors make proper diagnoses (17). In the classification of grade, the outcome of AI alone and doctor alone is not very good, but doctors’ prediction including the AI results improved the accuracy. In the classification of histology, both doctors and AI had high accuracy, but the accuracy of doctors combined with AI was improved. The same is true for staging. The accuracy of staging is not high, but doctors combined with AI improved the accuracy. Comparing with and without AI assistance, the time consumption for doctors with AI assistance is only 10 s longer, only 0.5 s per patient, 1.7% longer than before, which can be seen as almost no additional time cost. The extension of time consumption is not because of the speed predicted by AI, but because doctors need to analyze the information from AI.

Therefore, the AI model we built can effectively assist doctors in preoperative diagnosis and prediction of histology, stage, and grade.

There are several limitations to this study. Some multi-category classifications, such as staging and differentiation, have small sample sizes, resulting in poor overall performance. This was a single-center (country) study and an independent validation set from another country can make the results more convincing. Prospective, multi-center, large sample size research will help improve the performance of this AI model. In addition, the features of the database are mainly derived from text information, and the dimension of information should be improved. In the future, more dimensional information can be directly extracted from the images and examinations, so that intuitive information can be extracted.

## 5 Conclusion

This study demonstrated that a random forest model can predict histology, stage, and grade of endometrial cancer preoperatively and help doctors in obtaining a better diagnosis and predictive results with minimal additional time, which can help patients receive timely, appropriate, and effective treatment.

## Data Availability Statement

The original contributions presented in the study are included in the article/supplementary material. Further inquiries can be directed to the corresponding authors.

## Author Contributions

Conception and design: YF, ZW, HG, and SW. Development of methodology: YF, ZW, HG, SW, ZYZ, MX, JL, AT, and AD. Acquisition of data: YF, ZW, HG, SW, ZYZ, MX, and JL. Analysis and interpretation of data: YF, ZW, HG, SW, ZYZ, AT, and AD. Writing, review, and/or revision of the manuscript: YF, ZW, MX, JL, HG, BD, ZQZ, SW, YS, ZZ, AT, AR, CL, SX, AD, ZYZ, and LQ. Administrative, technical, or material support: YF, ZW, MX, HG, JL, ZZ, ZQZ, SW, BD, AR, CL, ZYZ,SX, YS, AD, AT, and LQ. Study supervision: YF, ZW, HG, SW, ZYZ, AT, AD, and LQ. All authors contributed to the article and approved the submitted version.

## Funding

This work was generously sponsored by Beijing Municipal Administration of Hospitals Clinical medicine Development of special funding-YangFan Project (Project No. ZYLX201713).

## Conflict of Interest

The authors declare that the research was conducted in the absence of any commercial or financial relationships that could be construed as a potential conflict of interest.

## Publisher’s Note

All claims expressed in this article are solely those of the authors and do not necessarily represent those of their affiliated organizations, or those of the publisher, the editors and the reviewers. Any product that may be evaluated in this article, or claim that may be made by its manufacturer, is not guaranteed or endorsed by the publisher.
